# Genomic epidemiology and public health implications of zoonotic monophasic *Salmonella* Typhimurium ST34

**DOI:** 10.3389/fcimb.2025.1490183

**Published:** 2025-03-11

**Authors:** Xiaolei Wu, Jiaxin Du, Xiao Zhou, Xianqi Peng, Chenghao Jia, Baikui Wang, Beibei Wu, Yan Li, Min Yue

**Affiliations:** ^1^ Department of Veterinary Medicine, Zhejiang University College of Animal Sciences, Hangzhou, China; ^2^ Institute of Tuberculosis Control, Zhejiang Provincial Center for Disease Control and Prevention, Hangzhou, China; ^3^ Hainan Institute of Zhejiang University, Sanya, China; ^4^ State Key Laboratory for Diagnosis and Treatment of Infectious Diseases, National Clinical Research Center for Infectious Diseases, National Medical Center for Infectious Diseases, The First Affiliated Hospital, College of Medicine, Zhejiang University, Hangzhou, China; ^5^ Key Laboratory of Systems Health Science of Zhejiang Province, School of Life Science, Hangzhou Institute for Advanced Study, University of Chinese Academy of Sciences, Hangzhou, China

**Keywords:** genomic epidemiology, monophasic *Salmonella* Typhimurium, sequence type 34, multi-drug resistance, public health, antimicrobial resistance

## Abstract

**Background:**

Monophasic *Salmonella* Typhimurium sequence type 34 (mSTM ST34) has emerged as a significant global health threat, but our understanding of its genomic epidemiology and potential public health implications in international and regional contexts remains limited. This study aims to fill this crucial gap by assessing the genomic epidemiology of multidrug resistance (MDR) mSTM ST34, as well as its clinical characteristics and virulence.

**Methods:**

To achieve the objectives of this study, we conducted a comprehensive genomic analysis of mSTM ST34 isolates. We obtained a global dataset comprising 13,844 strains from public databases, along with 339 strains from a regional surveillance collection in Zhejiang Province, China. This dataset aims to provide in-depth insights into antimicrobial resistance, mobile genetic elements, and pathogenicity. Additionally, we meticulously assessed the association between phenotypic profiles and clinical presentations.

**Results:**

Our findings revealed that the prevalence of mSTM ST34 has surpassed that of the previously dominant ST19. In addition, we observed an increase in the detection of the IncQ1 plasmid, which is responsible for disseminating MDR. The prevalence of mSTM ST34 carriage was exceptionally high among children (≤12 years old) and elderly individuals (≥65 years old), with 92.6% of the isolates exhibiting MDR, including resistance to frontline antimicrobials such as third-generation cephalosporins and ciprofloxacin. Additionally, the human mSTM ST34 strain demonstrates a remarkable capacity for biofilm formation, which increases its virulence in animal models and complicates therapeutic interventions.

**Conclusions:**

mSTM ST34 has surpassed the previously dominant ST19, and its ability to transmit across multi-species increases its potential for further human transmission. This study addresses critical gaps in our understanding of mSTM ST34 prevalence, highlighting the importance of whole genome sequencing in surveilling zoonotic pathogens.

## 1 Introduction

Non-typhoidal *Salmonella* (NTS) is a major global public health threat, significantly contributing to the burden of foodborne illnesses and outbreaks worldwide. It is estimated that NTS causes 93.8 million cases of gastroenteritis, leading to approximately 155,000 deaths each year globally (Majowicz et al., 2010). This substantial impact is exacerbated by the growing issue of bacterial antimicrobial resistance (AMR), which is a pressing global concern. The widespread dominance of certain NTS serovars and genotypes partly drives the proliferation of AMR. Consequently, AMR NTS infections threaten human health and impose severe economic losses on the global livestock and poultry industries (Ripon et al., 2023). Addressing this dual challenge is crucial for safeguarding both public health and global food safety.

NTS infections caused by the monophasic *Salmonella* Typhimurium sequence type 34 (mSTM ST34) are rapidly emerging as a severe public health issue, with an increasing risk of antimicrobial therapy failure worldwide (Sun et al., 2020). mSTM ST34 can be considered an evolved variant of the parent strain *Salmonella* Typhimurium (STM), characterized by several genomic deletions that have accumulated over time, leading to the replacement of the *fljB*-encoded flagellar antigen. This hallmarked change is often associated with distinct epidemiological profiles, including increased virulence and resistance to various classes of antimicrobial agents (Biswas et al., 2019; Ingle et al., 2021). Epidemiological investigations have identified chicken, pork, beef, salami, and chocolate as vehicles for mSTM ST34 around the world (Mossong et al., 2007; Raguenaud et al., 2012; Cito et al., 2016; Andreoli et al., 2017; Lund et al., 2022). In China, mSTM ST34 has seen a dramatic spread in recent years, with frequent detection in food samples, environmental and human specimens (Elbediwi et al., 2020; Zhang et al., 2022; Nambiar et al., 2024). Notably, mSTM ST34 has exhibited a remarkable ability to acquire resistance to multiple classes of antimicrobial agents, including critically important antimicrobials in human medicine, such as fluoroquinolones, third-generation cephalosporins, extended-spectrum β-lactams, and polymyxins (Qin et al., 2022). Collectively, the combination of horizontal gene transfer, and co- and cross-resistance mechanisms could facilitate the rapid spread of resistance determinants within bacterial populations (Christaki et al., 2020), posing challenges to current established therapeutic options (Yang et al., 2017; Sun et al., 2020). Indeed, the burden of multidrug-resistant (MDR) mSTM ST34 has increased with the complications of post-COVID-19 relaxation (Huang et al., 2024).

In recent years, adopting whole-genome sequencing (WGS) technology has revolutionized our understanding of pathogen evolution and epidemiological investigations. WGS offers unparalleled high-resolution for characterizing genetic diversity, tracing transmission routes, and identifying evolutionary relationships among bacterial isolates. By comparing whole genomes, researchers can elucidate mechanisms underlying the emergence and spread of pathogens, including the acquisition of antimicrobial resistance genes and virulence determinants (Wren, 2003; The et al., 2016). Additionally, WGS facilitates detecting outbreak clusters, enabling timely public health interventions to mitigate further transmission (Deng et al., 2021). This technology provides valuable insights into the geographic distribution, population structure, and evolutionary dynamics of prevalent pathogens. Furthermore, WGS-based surveillance enables monitoring genetic changes over time, identifying emerging threats, and informing targeted control strategies (Jia et al., 2023; Zhou et al., 2023).

Here, we used genome sequencing, phenotypic profiling, and epidemiological data to systematically assess the trend, pathogenicity, and drug resistance of mSTM ST34 in both global and regional contexts. We discovered that mSTM ST34 has eclipsed the previously dominant ST19. Its broad host range increases the potential for mSTM ST34 to transmit to humans, and its high pathogenicity significantly impacts the poultry product chain while posing a severe threat to human health. This is particularly concerning due to the high prevalence of MDR in vulnerable populations, such as children and the elderly. This study fills gaps in our understanding of the prevalence of mSTM ST34 and provides a scientific basis for preventing and mitigating these infections.

## 2 Materials and methods

### 2.1 Bacterial strains

Both the mSTM ST34 isolates from poultry (Sm78, Sm103) and the STM ST19 strain SL1344 were obtained from our lab’s collection. mSTM ST34 strain SSH006 was isolated from a female patient who showed resistance to imipenem and cephalosporin, two of the most often tested antimicrobial agents (Yang et al., 2017).

### 2.2 Bacterial genomes and data sources

To achieve the objectives of this study, searches were conducted in the Enterobase databases to retrieve the genomes of STM and mSTM using the serovar option (“Typhimurium” OR “i:” OR “monophasic”) (Zhou et al., 2020). Complete genome sequences of *Salmonella* were obtained from National Center for Biotechnology Information (NCBI, https://www.ncbi.nlm.nih.gov/). Additional data were sourced from the China National GeneBank Database under BioProject accession CNP0004753.The search strategy was followed to ensure that the released genome around the globe before February 2023 related to the prevalence of mSTM ST34 should be collected and included in this systematic research for further processing and screening. The complete information was summarized in **Supplementary Table S1**.

Additionally, from 2019 to 2022, the Centers for Disease Control and Prevention and general hospitals collaborated on a clinical surveillance program for *Salmonella* infections in patients from Zhejiang Province, China. A total of 1,270 patients, aged 0 to 92 years, were clinically confirmed with a *Salmonella* infection diagnosis. Fecal samples were cultured according to the recommended protocol (Mikoleit, 2010). Diarrhea was defined as having three or more abnormally loose stools in the previous 24 hours. All isolated strains were serotyped by slide agglutination using commercial antiserum (SSI Diagnostica, Denmark). No sex preference was considered when recruiting patients. Demographic information, medical history, and both typical and atypical symptoms of *Salmonella* infection were provided by participants upon recruitment, and clinical data during hospitalization were collected from electronic medical records. Detailed information is provided in **Supplementary Table S2**.

### 2.3 Whole genome sequencing

The Gram-negative bacterial genomic DNA extraction kit (DR0306050; Easy-Do) was used to extract DNA from isolates. Sequencing was performed using an Illumina Novaseq 6000 platform in 150 bp paired-end mode. *De novo* assembly was conducted by SPAdes v.3.12.0, as previously reported (Bankevich et al., 2012). All detected genomes have been deposited in the China National GeneBank DataBase under BioProject Accession CNP0004753.

### 2.4 Genomic data analysis

All isolates were confirmed by *in silico* serotyping conducted by SISTR v.1.1.1 (Yoshida et al., 2016). Subsequently, multi-locus sequence typing (MLST) was performed using MLST v.2.19.0 (https://github.com/tseemann/mlst). The antimicrobial resistance genes were identified using ResFinder v.4.1 (>90% identity and >80% coverage) (Bortolaia et al., 2020). The plasmids were identified using MOB-suite (Robertson and Nash, 2018).

SNPs in the core genome were identified by Snippy v.4.4.5 (https://github.com/tseemann/snippy). The recombination regions were filtered from the core genome alignment by Gubbins v.2.3.4 (Croucher et al., 2015). The maximum-likelihood phylogenetic tree was generated using the best model (TVM+F) by IQ-TREE v.1.6.12 (Nguyen et al., 2015). TreeTime provides routines for ancestral sequence reconstruction and inference of molecular-clock phylogenies (Sagulenko et al., 2018).

In addition, the invasiveness index was assessed according to the method described previously (Wheeler et al., 2018). Briefly, we used the random forest model trained by the former method and R v.4.1.2 to calculate the invasiveness index. This model showed the applicability of calculating the invasiveness index for different species of *Salmonella*. Specifically, the DeltaBS metric was used to identify mutations in protein-coding genes in the whole genome sequence of the strains used for model building, from which 196 top predicted genes were obtained for measuring invasiveness in *S*. *enterica*.

### 2.5 Antimicrobial susceptibility testing

Antimicrobial susceptibility testing was performed using the broth dilution method. The minimal inhibitory concentrations (MIC) of eight antimicrobial agents—ampicillin, amoxicillin/clavulanic acid, gentamicin, tetracycline, spectinomycin, streptomycin, trimethoprim-sulfamethoxazole and meropenem—were determined. The results were interpreted according to Clinical and Laboratory Standards Institute guidelines (Patel, 2016). *Escherichia coli* ATCC 25922 was used as a quality control. Isolates that showed resistance to at least three classes of antimicrobials were defined as MDR.

### 2.6 Bacterial growth curve

A 1:100 dilution of a saturated culture was incubated overnight in LB or M9 medium (6 g/L Na_2_HPO_4_, 3 g/L KH_2_PO_4_, 1 g/L NH_4_Cl, 0.5 g/L NaCl, 2 g/L glucose, 246.5 mg/L MgSO_4_·7H_2_O, 1 mg/L thiamine·HCl, 14.7 mg/L CaCl_2_, pH 7 ± 0.2 at 25°C) with shaking at 180 rpm at 37°C. The OD_600_ was measured at different time points. To rescue SSH006 growth, casamino acids (CAS: 65072-00-6;C304284; Aladdin; Shanghai, China) were added to the culture at 4 h.

### 2.7 Motility assay

The overnight culture was diluted to 0.06–0.08 of OD_600_. Three microliters of the bacterial suspension were added to the center of LB medium plate containing 0.3% agar. The plates were incubated statically at 37°C for 6 h under aerobic and anaerobic conditions. The displacement was measured via a vernier caliper at 6-hour intervals. Three replications were performed for each group.

### 2.8 Survival in phosphate-buffered saline

The strain was washed and resuspended in 1 × phosphate-buffered saline (PBS). A bacterial suspension equivalent to ~10^7^ colony-forming unit (CFU)/ml was added to PBS and incubated at 180 rpm at 37°C. The number of colonies and the OD_600_ were measured every day for nine days. Relative survival was calculated as the ratio of CFU at each time point to CFU at time zero. At least three independent trials were performed.

### 2.9 Virulence assay of mSTM ST34 isolates in animals

Specific pathogen-free Babcock chicken embryos were obtained from Shennong Chick Farm. The 19-day-old embryos were hatched under biosecurity conditions in a 37.2°C incubator. Chicks were fed under aseptic conditions until 1 day of age. Water and food were withheld for 3 hours before and after gavage with mSTM ST34 isolates or SL1344 at a concentration of 10^8^ CFU/mL. Chicken survival (n=17~37 per group) was monitored daily for 7 days. At 2 and 5 days post-infection (dpi), the heart, liver, and spleen (n=3, per group) were weighed and homogenized in PBS. Samples were serially diluted and dispensed onto xylose lysine deoxycholate (XLD) agar to count the number. Feces were collected during the 7-day monitoring period.

Six to eight-week-old female C57BL/6N mice were purchased from Vital River Laboratory Animal Technology (Beijing, China). The mice were maintained under a 12-hour light/dark cycle and fed a standard chow diet in a specific pathogen-free (SPF) facility at the Laboratory Animal Center of Zhejiang University, following the National Institutes of Health Guide for the Care and Use of Laboratory Animals. The streptomycin-pretreated mouse colitis model was described in a previous study (Barthel et al., 2003). Water and food were withheld for 4 hours before gavage with 20 mg of streptomycin, after which they were reintroduced. Twenty hours later, water and food were again withheld for 4 hours. Single colonies were inoculated to an OD600 of ~0.1 (~10^8 CFU/mL) and then diluted 100-fold. The mice were infected intragastrically with 100 μL of the bacterial suspension. Water and food were provided 1 hour post-infection. At 3 and 5 dpi, the infected mice were sacrificed, and tissue samples were collected. Tissues were homogenized in PBS buffer, and CFU were determined by serial dilution plating on XLD solid plates containing 100 μg/mL streptomycin or 100 μg/mL streptomycin plus 50 μg/mL ampicillin. Samples that fell below the detection limit were recorded as 0 CFU.

For histopathology staining, the tissue samples fixed in 4% paraformaldehyde (BL539A; biosharp; Hefei, China) were embedded in paraffin, sliced (4 μm thickness), dehydrated and then sectioned for hematoxylin and eosin (HE) staining.

### 2.10 Rdar phenotype

The Rdar morphology was determined as previously described (Singletary et al., 2016). The 3 μL of ~100 CFU/ml bacterial solution was dripped in lysogeny broth (LB) plates without salt and supplemented with 40 μg/mL Congo red (CAS: 573-58-0; Aladdin; Shanghai, China) and 20 μg/mL Coomassie blue (CAS: 6104-58-1; Aladdin; Shanghai, China). Plates were incubated at 37°C for 5 days without inversion. To ensure accurate results, duplicates of every experiment were conducted.

### 2.11 Ethics statement

Ethical approval was granted by the Ethics Committee of the Zhejiang Centers for Disease Control and Prevention (2019-014). No potentially identifiable images or data were included in this article, and the data cannot be used to identify individuals. Written informed consent for participation was not required for this study in accordance with national legislation and institutional requirements. All experimental protocols (ZJU20190093; ZJU20220295) involving animals were conducted according to the ethical policies and procedures approved by the Review Committee of the College of Animal Sciences at Zhejiang University.

### 2.12 Data analysis and visualization

Quantitative data, exponential (Malthusian) growth, and Pearson correlation were supported by GraphPad Prism 9. All statistical details of experiments, including statistical tests, the exact value of n, what n represents, the p-value and the means with standard deviations, were provided in the corresponding figure legends. Figure layout and proper annotation were accomplished by Adobe Illustrator 2021 (https://www.adobe.com). Schematics were created using BioRender (https://biorender.com).

## 3 Results

### 3.1 Rapid dissemination of mSTM ST34 over the past decades

**Figure 1:**
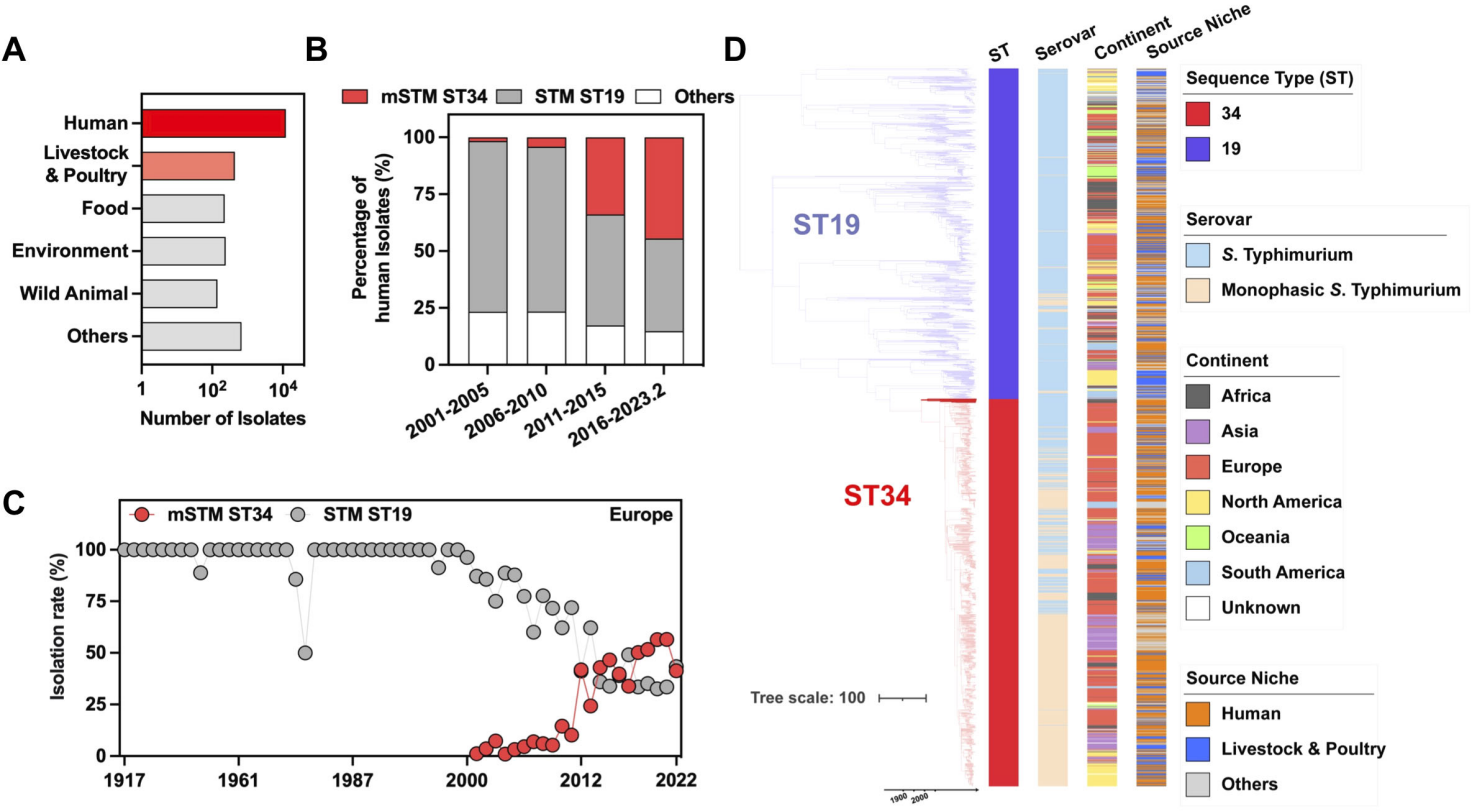
Prevalence of human monophasic *Salmonella* Typhimurium sequence type 34 (mSTM ST34). **(A)** The total number of examined isolates among different sources. Humans, livestock and poultry are the primary sources of isolation for mSTM ST34. **(B)** The number and percentage of global human isolates among different types show a rapid expansion in ST34, particularly mSTM ST34 (highlighted in red). STM indicates *Salmonella* Typhimurium, while ST19 or ST34 refers to specific genotypes designated as sequence types. **(C)** The prevalence dynamics of European mSTM ST34 (red) and STM ST19 (grey) are shown. From 2001 onward, the overall growth rate of STM ST19 was lower than that of mSTM ST34. **(D)** Bayesian evolutionary analysis presents the maximum-clade credibility tree of ST34 (n = 1,091) and ST19 (n = 931) isolates, illustrating the timing of lineage divergence and genetic changes. The time in years is indicated on the x-axis. The accompanying heatmap displays the sequence types, serovars, and isolation sources of the isolates.

To project a global picture of mSTM ST34, we included the analysis of a total of 44,282 isolates from lab collections and various databases (**Supplementary Table S1**). Most mSTM ST34 isolates are from humans, followed by poultry and livestock (**Figure 1A**). We further examined the mSTM ST34 isolates from humans. Over the past two decades, the prevalence of mSTM ST34 showed a significant increase from 1.7% in 2001-2005 to 44.5% in 2016-February 2023 (**Figure 1B**). This growth was most noticeable in Europe. STM ST19 persisted among human isolates in the 1990s, but from 2001 onward, the overall growth rate of STM ST19 was lower than that of mSTM ST34 (**Figure 1C**). Spatiotemporal evolution analysis showed that the ST34 strain formed a distinct branch, separating it from ST19, and mSTM ST34 derived from STM ST34 introduced into different continents and set off a global pandemic that affected livestock, poultry, and humans (**Figure 1D**). Overall, the widespread distribution of mSTM ST34 poses a significant challenge for livestock and poultry production as well as clinical treatment.

### 3.2 Genotypic antimicrobial resistance of mSTM ST34 and associated plasmids

**Figure 2:**
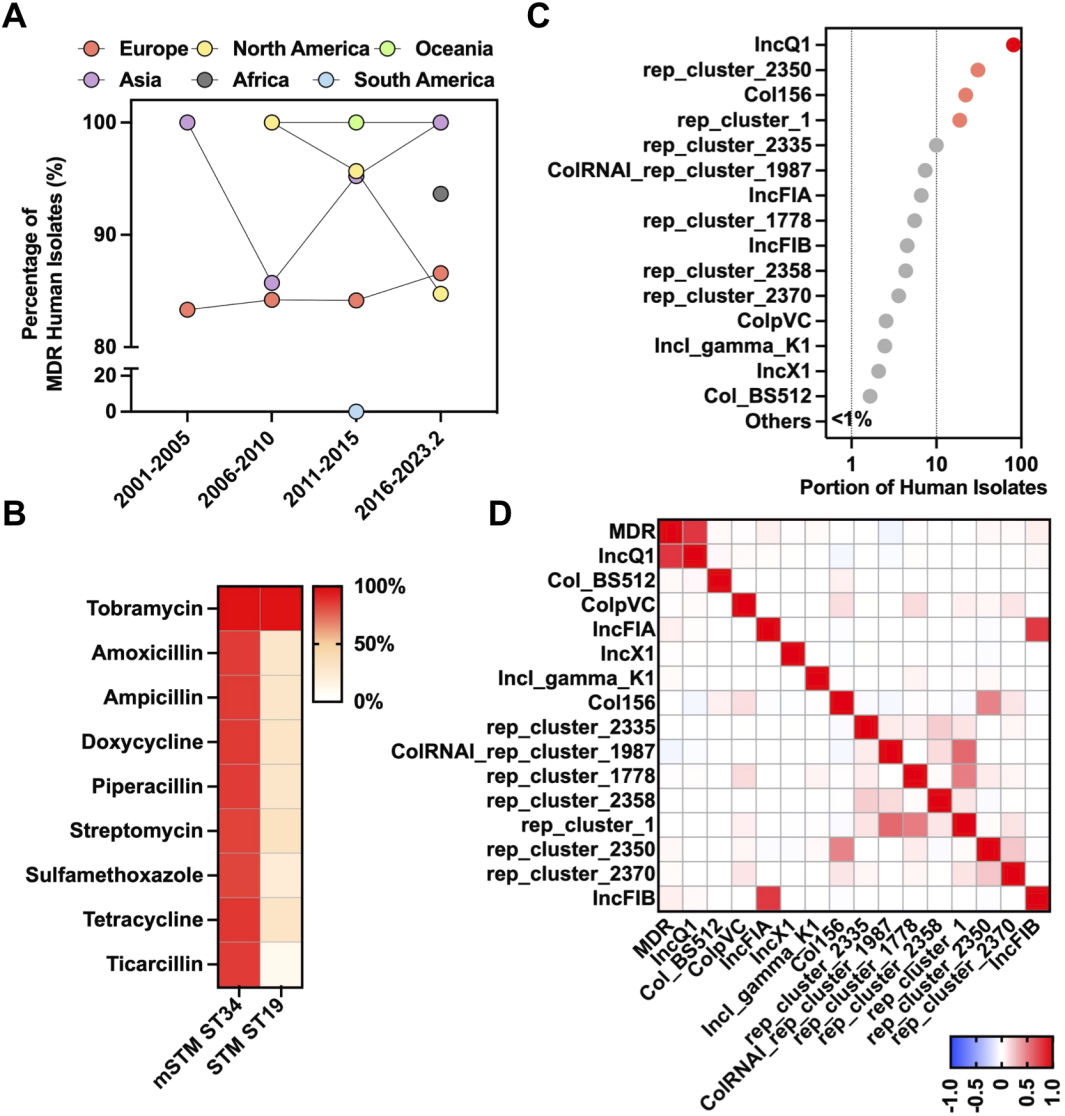
Genotypic antimicrobial resistance of mSTM ST34. **(A)** Temporal dynamics and geographical distribution of global multidrug resistance (MDR) mSTM ST34 in humans. In general, more than 80% of isolates from most continents across the examined periods, except South America between 2001 and February 2023, showed the MDR phenotype. **(B)** A comparison of antimicrobial-resistant patterns between human mSTM ST34 and STM ST19 isolates. The antimicrobial resistance potential is predicted by the whole-genomic sequence dataset based on ResFinder. A red-white colour gradient indicated the percentage of isolates. **(C)** A total of 116 conjugative plasmid types of human mSTM ST34 isolates are predicted by the whole-genomic sequence dataset via MOB-suite. **(D)** The correlation heatmap of MDR with conjugative plasmid types (≥1% of isolates harbour). The IncQ1 was positively associated with the MDR phenotype among human mSTM ST34 isolates. The scale from blue to red shows a Pearson correlation from -1 to 1, indicating a negative to positive association.

The whole genome sequences were screened for the detection of antimicrobial determinants encoding resistance to various antimicrobial categories. Our findings revealed a steady high prevalence of MDR mSTM ST34 worldwide during the past two decades, with 80% to 100% of the isolates showing MDR (**Figure 2A**). Compared to STM ST19, mSTM ST34 isolates showed higher resistance to several classes of antibiotics, including aminoglycosides (tobramycin, 99.7%; streptomycin, 83.8%), β-lactams (amoxicillin, 85.3%; ampicillin, 85.6%; piperacillin, 85.2%; ticarcillin, 85.2%), sulfonamides (sulfamethoxazole, 83.1%), and tetracyclines (doxycycline, 86.2%; tetracycline, 86.6%) (**Figure 2B**). Additionally, we revealed that the mSTM ST34 population contains 116 conjugative plasmid types (**Figure 2C**). Notably, conjugative IncQ1 showed a high correlation with the MDR pattern of mSTM ST34 (Pearson correlation, 0.781) (**Figure 2D**).

### 3.3 Clinical characteristics of mSTM ST34 infections

**Figure 3:**
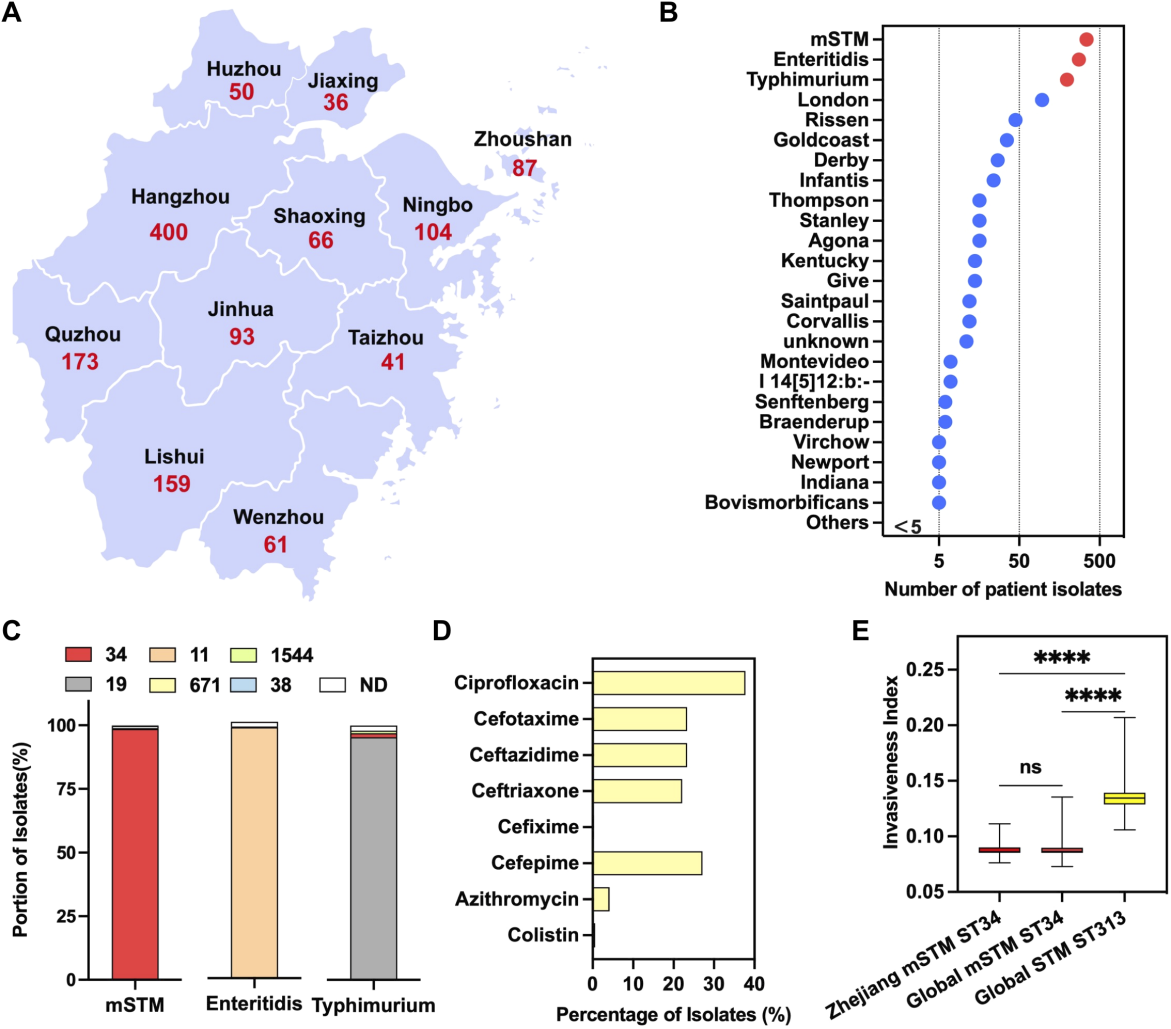
A local surveillance of human Salmonellosis in Zhejiang Province, China. **(A)** A geographical distribution of confirmed *Salmonella*-infected cases, from 2019 to 2022 (n=1,270), in each city of Zhejiang Province. **(B)** Ranking the *Salmonella* serovars for human infections. The red dot indicates three common serovars, including monophasic *Salmonella* Typhimurium (mSTM), Typhimurium and Enteritidis. The *Salmonella* remaining serovars were marked in blue. **(C)** The genotype of *Salmonella* serovars for human infections, with the number indicating a specific sequence type (ST). More than 98.5% of the mSTM isolates are ST34 (in red). **(D)** The percentage of mSTM ST34 isolates resistant against clinically relevant antimicrobials, including ciprofloxacin, azithromycin, colistin, third-generation cephalosporins (cefixime, cefotaxime, ceftazidime and ceftriaxone) and fourth generation cephalosporin, cefepime. **(E)** A comparison of the invasiveness index between local clinical and global clinical isolates. Compared with the high invasiveness index of ST313 lineage isolates, there is a similar invasiveness index between clinical isolates from Zhejiang and global clinical isolates. P values were calculated by using a nonparametric test (Kruskal-Wallis test). ns, no significant; ∗∗∗∗, p<0.0001.

From April 2019 to September 2022, 1270 patients infected with *Salmonella* were identified from hospitals in Zhejiang Province, China (**Figure 3A**). The main serovars included mSTM (344 isolates), Typhimurium (276), Enteritidis (196) (**Figure 3B**). The dominant subtype of mSTM isolates was ST34 (339/344), which was included in this study cohort (**Figure 3C**). The complete information about these patients was summarized in **Supplementary Table S2**. The surveillance results showed that 153 children (≤12 years old) and 20 elderly (≥65 years old) were infected with MDR mSTM ST34. One outbreak affected seven patients (24~45 years old) in a hospital in Quzhou City. Notably, genomic analysis revealed that some isolates are resistant to clinical antibiotics, including ciprofloxacin (37.8%), third-generation cephalosporins [cefixime (0.3%); cefotaxime (23.3%); ceftazidime (23.3%); ceftriaxone (22.1%)], fourth-generation cephalosporin [cefepime (27.1%)], azithromycin (4.1%) and colistin (0.6%) (**Figure 3D**).

Consistent with global isolates, compared with highly invasive STM ST313, these mSTM ST34 isolates have a lower invasiveness index (**Figure 3E**), indicating that mSTM ST34 mainly maintains an intestinal lifestyle. This corresponds to most patients having diarrhea symptoms (295/339) instead of septicemia (**Supplementary Table S2**).

### 3.4 Phenotypic profiling of the mSTM ST34 isolates

**Figure 4:**
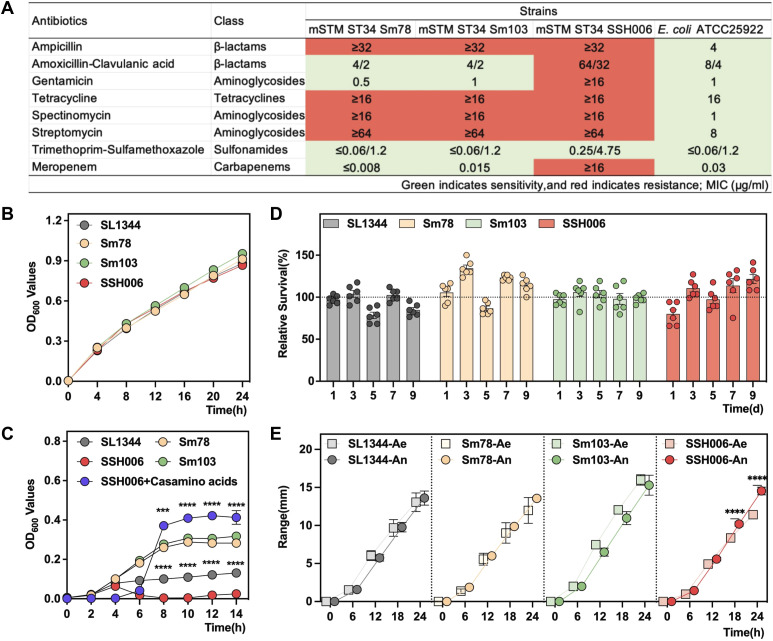
Phenotypic characteristics of the mSTM ST34 isolates. **(A)** The resistance of mSTM ST34 isolates and the reference strain *E coli* ATCC 25922 to the tested antimicrobial agents from five classes indicates that mSTM ST34 isolates exhibit multi-drug resistance, particularly to ampicillin, tetracycline, spectinomycin, and streptomycin. Overall, the level of drug resistance in the human strain SSH006 is notably serious. **(B, C)** Growth curves of SL1344 (grey), Sm78 (orange), Sm103 (green) and SSH006 (red) in LB **(B)** and M9 **(C)** media. The SSH006 strain grew poorly in the M9 medium, but this effect was alleviated by supplementation with casamino acids (purple). The difference between SSH006 and SL1344, as well as between SSH006 and SSH006+ casamino acids, was assessed using a two-way ANOVA test. **(D)** Survival of the four strains in PBS for 9 days. mSTM ST34 isolates were able to persist in a nutrient-poor PBS solution, and its growth condition recovered on day 9. **(E)** Motility assay under aerobic (Ae) and anaerobic (An) conditions. A two-way ANOVA test was conducted to determine whether there is a significant difference in the motility of SSH006 under aerobic and anaerobic conditions. Compared with aerobic condition, SSH006 was more motile under anaerobic conditions. **(B, D)**. ∗∗∗, p<0.001; ∗∗∗∗, p<0.0001. A-C, n=6; D, n=3.

In this study, we compared the traits of mSTM ST34 and STM ST19, represented by multi-drug resistant mSTM ST34 isolates Sm78, Sm103, SSH006 (**Figure 4A**), alongside the STM ST19 isolate SL1344. The four isolates grew rapidly in LB medium and did not show any difference (**Figure 4B**). However, in the nutrient-poor M9 medium, SSH006 was barely able to grow in the M9 medium (**Figure 4C**). The complement of casamino acids rescued the growth of SSH006. Despite the poor growth in M9 medium, SSH006 was able to persist in a nutrient-poor PBS solution, and its growth condition was recovered on day 9 (**Figure 4D**). mSTM ST34 only expressed *fliC*-encoded flagella, but had no defect in motility (**Figure 4E**). In addition, SSH006 was more motile under anaerobic conditions than under aerobic conditions.

**Figure 5:**
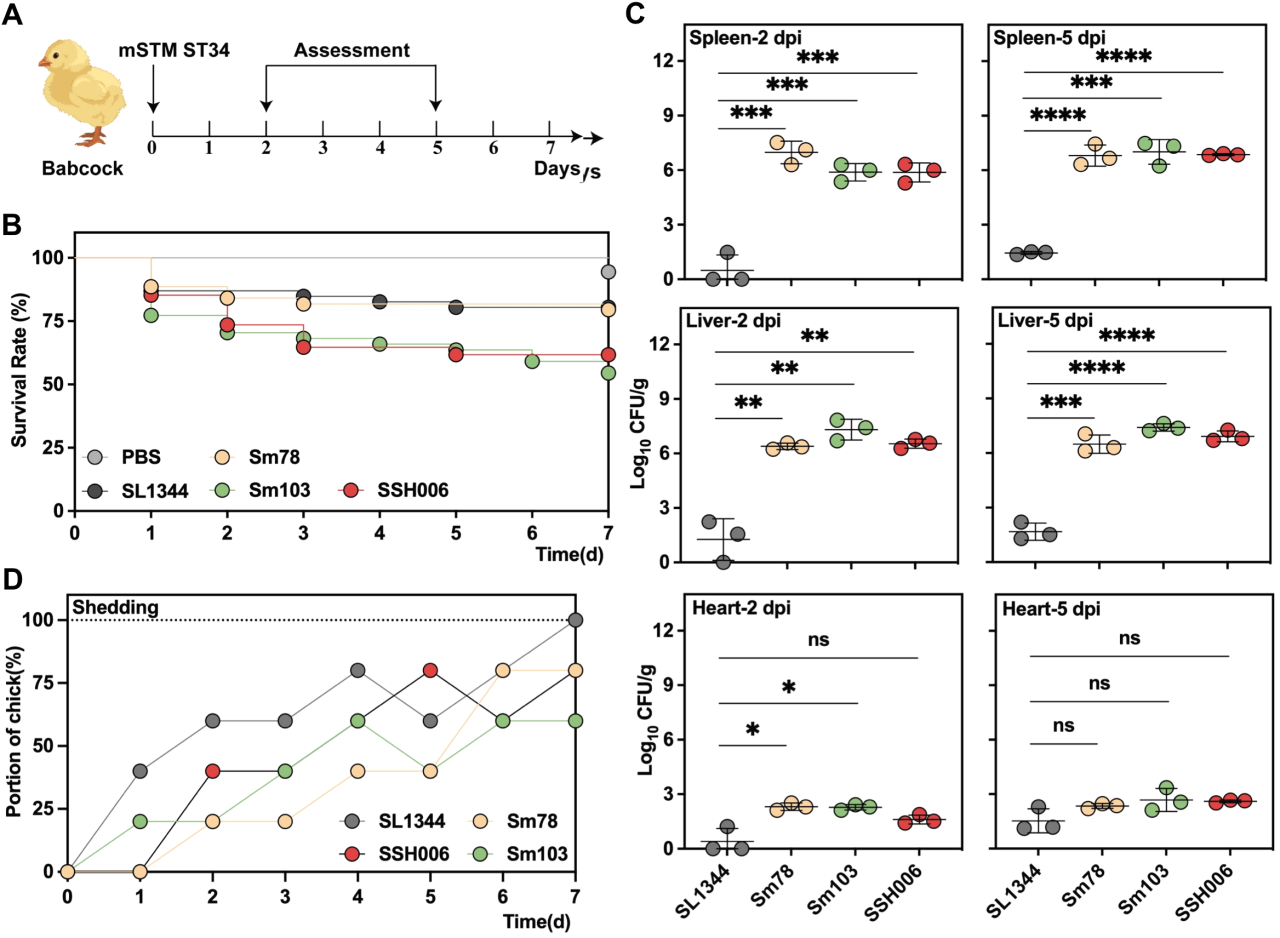
The highly virulent and transmissible mSTM ST34 poses a significant threat to the poultry industry. **(A)** Virulence assessment based on a Babcock chick infection model. 1-day old chicks, collected from SPF embryos with a negative test for *Salmonella*, were used in the assay. **(B-D)** Three mSTM ST34 isolates (Sm78 in orange, Sm103 in green and SSH006 in red), and one reference isolate (SL1344 in grey), indicating as STM, were used. **(B)** Survival curves for the examined isolates during a 7-day examining period. The chicks infected with mSTM ST34 isolates showed the lower survival rate (Sm103, 54.5%; SSH006, 61.8%; Sm78, 79.5%), compared to SL1344 (80.4%), and PBS (100%, in white) as a negative control. n=17~37. **(C)** The bacterial loads were determined by serial-dilution plating on XLD agar plates. The higher bacterial burden in in groups infected with mSTM ST34 isolates was determined at day 2 (left) or 5 (right) post-infection. CFU, colony-forming unit. Statistical significance was determined by a t test. ns, not significant; ∗, p<0.05; ∗∗, p<0.01; ∗∗∗, <0.001; ∗∗∗∗, p<0.0001. n = 3. **(D)** Feces were collected from the chicks at the indicated time points. Continuous shedding of mSTM ST34 was observed throughout the 7-day monitoring period. The dots represent the proportion of positive samples within each group.

The virulence capacity of the mSTM ST34 isolates was first assessed by *in vivo* chick infection (**Figure 5A**). The chicks infected with mSTM ST34 isolates showed the lower survival rate (Sm103, 54.5%; SSH006, 61.8%; Sm78, 79.5%), compared to SL1344 (80.4%) (**Figure 5B**). The infection feature assessed at 2 and 5 dpi showed a higher bacterial burden in the mSTM ST34-infected groups (**Figure 5C**). Additionally, continuous shedding of mSTM ST34 was observed throughout the 7-day monitoring period” to “observed throughout the 7-day monitoring period (**Figure 5D**).

**Figure 6:**
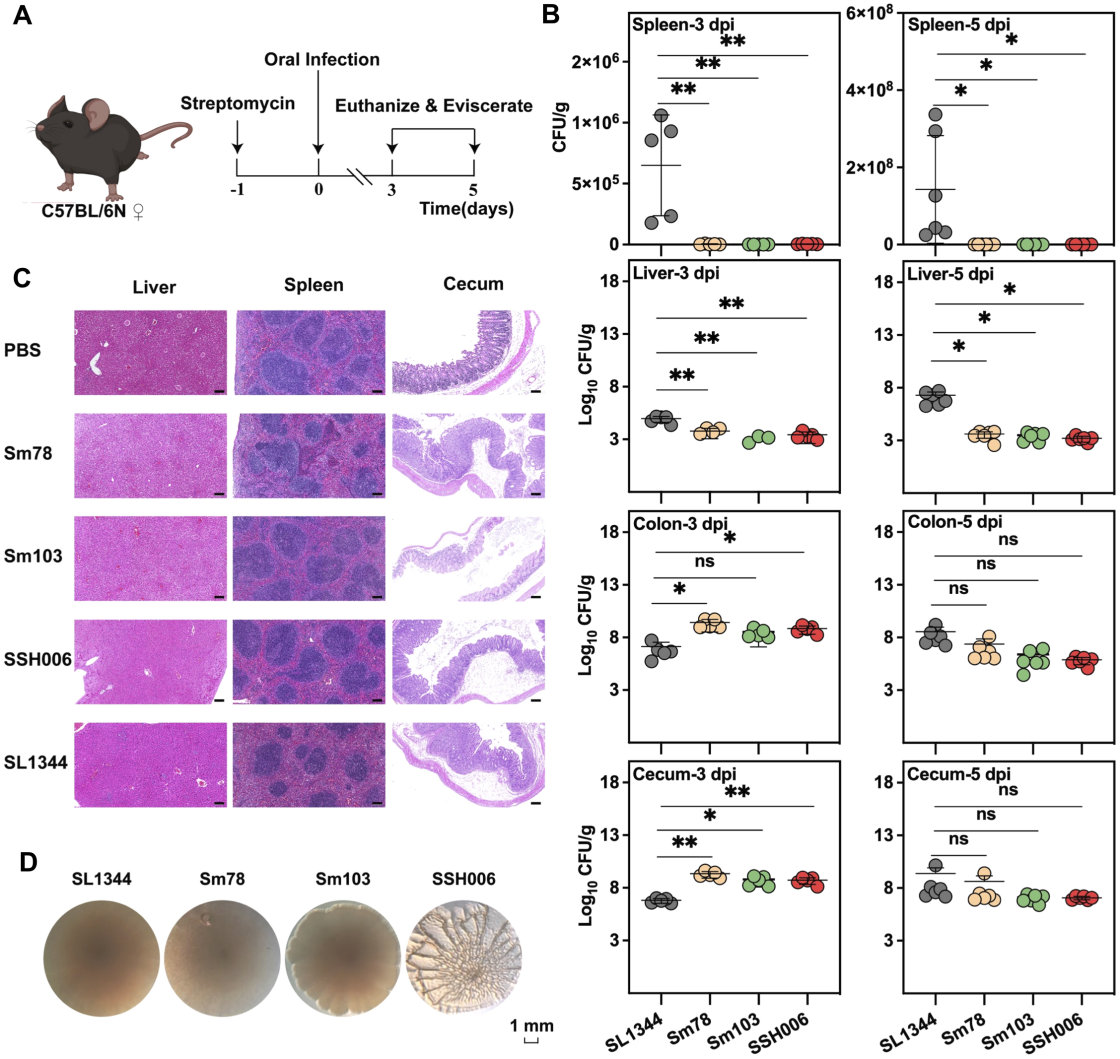
The mSTM ST34 is a pathogen that primarily colonizes the intestine. **(A)** Virulence assay in a streptomycin-treated mouse colitis model. **(B)** Colonization in systemic and intestinal sites is depicted. The bacterial load in various infection groups at day 3 (left) and day 5 (right) post-infection is represented as CFU/g, with each individual dot indicating the value. The mSTM ST34 isolate demonstrated a stronger colonization capability in the colon and cecum, while showing weaker colonization in the spleen and liver. Statistical significance was determined by a t test. ns, not significant; *, p<0.05; **, p<0.01. n = 5~6. **(C)** Representative images of tissues at 3 dpi between mSTM ST34-infected and SL1344-infected mice (4 μm descending section stained with H&E; Scale bar, 300 μm). The cecum in mSTM ST34 isolates-infected groups inflicted more severe damage. **(D)** Rdar phenotype showed that SSH006 has pronounced three-dimensional structure with elongated cells expressing curli and cellulose, while SL1344, Sm78 and Sm103 showed a smooth colony with a white rim.

We further utilized a streptomycin-pretreated mouse model to evaluate infection phenotypes (**Figure 6A**). The bacterial loads in the liver, spleen, colon, and cecum were determined at 3 and 5 dpi (**Figure 6B**). In comparison to the systemic typhoid-like infection SL1344 group, mSTM ST34 isolates exhibited a preference for intestinal colonization. Specifically, mSTM ST34 isolates were not detected in some spleen samples (8 out of 15), and the loads of mSTM ST34 in the colon and cecum were significantly higher than those of SL1344 at 3 dpi. Pathological sections also indicated that the cecum, rather than the liver and spleen, suffered more severe damage in the mSTM ST34 isolate-infected groups at 3 dpi (**Figure 6C**).

**Figure 7:**
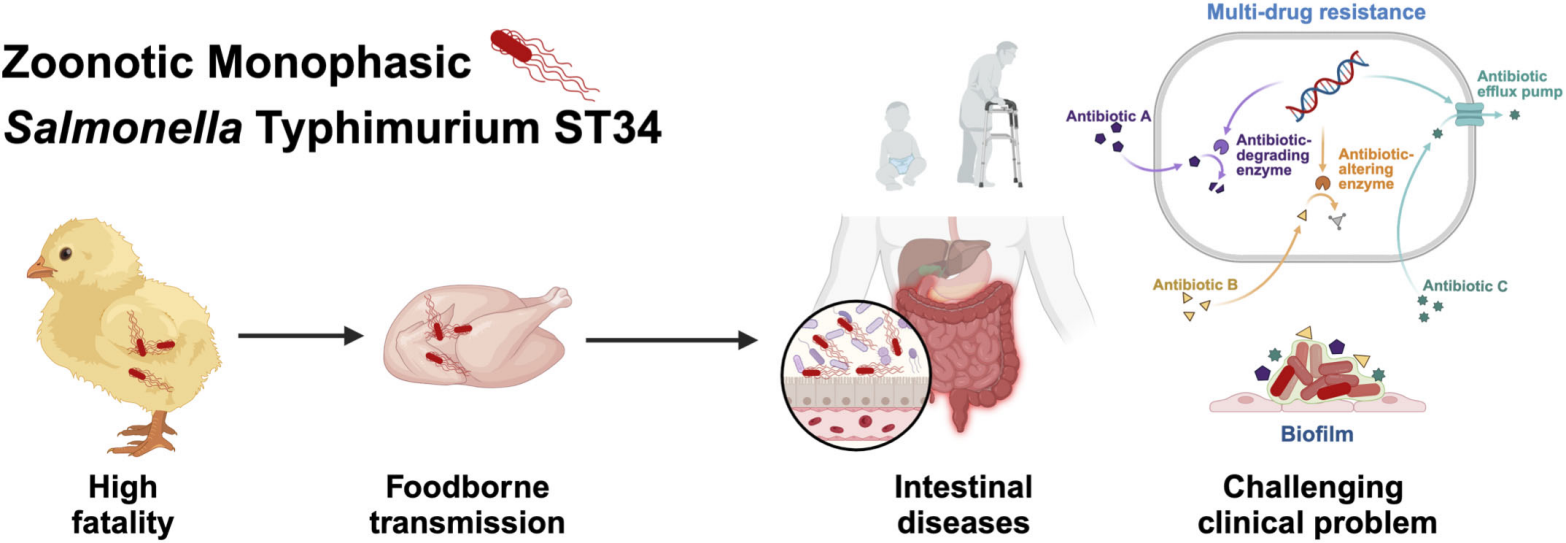
mSTM ST34 is a pathogen that has a significant impact on public health and the poultry product chain. In poultry, mSTM ST34 infections can lead to high mortality rates, posing a serious threat to animal health and food production. mSTM ST34 is also transmitted through foodborne routes and can survive and establish itself in the human intestine, resulting in intestinal diseases that typically manifest with symptoms such as diarrhea. Moreover, its multiple-drug resistance and persistent biofilm formation complicate clinical treatment, especially in vulnerable populations, including young children and the elderly.

Notably, mSTM ST34 SSH006 isolated from a patient showed substantial biofilm formation (**Figure 6D**). The mature biofilm on the Congo red plate had a distinct three-dimensional structure, with elongated cells expressing curli and cellulose. Biofilm formation protects mSTM ST34 from antibiotics and complicates clinical treatment. Overall, mSTM ST34 is a zoonotic pathogen that causes high mortality in poultry and leads to intestinal diseases, posing significant challenges for poultry production and clinical treatment (**Figure 7**). We observed considerable strain-specific dynamic in virulence and other phenotype.

## 4 Discussion

In this study, the global genomic analysis revealed that mSTM ST34 had circulated worldwide for decades and outcompeted the previously prevalent lineage, STM ST19. The competitive advantage of mSTM ST34 is also supported by findings from other studies (Naberhaus et al., 2019; Wang et al., 2023). mSTM ST34 has undergone different patterns of *fljB* loss (Arrieta-Gisasola et al., 2020), which may promote the invasion of epithelial cells, resulting in increased inflammation in the colon (Smith et al., 2003). Similarly, earlier research found that attenuated, less invasive flagellar mutants resulted in fewer granulocytes on the intestinal mucosa, allowing for greater *Salmonella* invasion (Methner et al., 2010). Furthermore, we showed that SSH006 strain changed metabolic genes and lacked the ability to manufacture some nutrients on its own, leading to poor growth in nutrient-deficient conditions, such as M9 medium. Previous research revealed that an African *Salmonella* strain had lost necessary genes for environmental stress resistance, indicating an adaptation to a human-to-human transmission (Singletary et al., 2016). Therefore, the metabolic or flagellar deletion could reflect the evolutionary process of mSTM ST34 adaptation to host.

mSTM ST34 is emerging as a significant threat in the livestock and poultry sectors (European Food Safety Authority and European Centre for Disease Prevention and Control, 2021). The results of animal infection showed that mSTM ST34 isolates were more lethal to chicks compared to SL1344 strain. This heightened virulence suggests that mSTM ST34 inflicts greater economic damage on the poultry industry, exacerbating the challenges faced by breeders. Furthermore, by infecting food-producing animals, mSTM ST34 gains additional opportunities for onward transmission to humans, leading to severe infection disorders. These infections are characterized by severe clinical outcomes that can range from mild gastroenteritis to severe invasive disease (Yang et al., 2017) and even deaths (Mossong et al., 2007), posing a particular risk to vulnerable populations such as children and the elderly.

The rising incidence of MDR mSTM ST34 represents a significant health crisis for healthcare systems globally (Sun et al., 2020). This MDR is often associated with the presence of the conjugative plasmid IncQ1, which carries multiple resistant determinants (McMillan et al., 2020). A recent study has highlighted the role of the small IncQ1 plasmid in transferring KPC-2 carbapenemase genes to invasive NTS strains (Fontana et al., 2021). Currently, cephalothin and ciprofloxacin are the most commonly antimicrobial drugs used to treat salmonellosis and lead to good clinical outcomes. However, the widespread resistance of mSTM ST34 to these antimicrobial drugs highlights a critical issue in managing infections. Hence, the growing resistance underscores the urgent need for the development of antimicrobial strategies and alternative treatment options. Phages can effectively lyse bacteria, providing a targeted approach to infection treatment while minimizing harm to beneficial microbes (Principi et al., 2019). Additionally, certain bacterial pathogens, including mSTM ST34, often replicate within human cells (Venturini et al., 2022). Consequently, many commonly used antimicrobials cannot penetrate human cells, rendering them ineffective against intracellular pathogens (Buccini et al., 2020). In contrast, phages could prove valuable for such treatments. Furthermore, phage therapy may help reduce the selective pressure that contributes to the emergence of antibiotic resistance (Lin et al., 2017). Phage therapy has been utilized in medicine for nearly 70 years in certain European countries, resulting in a wealth of clinical data (Rohwer and Segall, 2015). Recent advancements in phage therapy include preclinical testing in animal models, compassionate use in severely ill patients, and several clinical studies (Gordillo Altamirano and Barr, 2019; Gordillo Altamirano et al., 2022; Liang et al., 2023). Thus, phage treatment appears to be a promising alternative therapeutic approach for multidrug-resistant mSTM ST34. Additionally, probiotics are considered an alternative strategy for controlling *Salmonella* infectious diseases. They can enhance colonization resistance and immune defense, inhibit the colonization and invasion of pathogens, and help maintain gastrointestinal homeostasis (Gareau et al., 2010; Kotzampassi and Giamarellos-Bourboulis, 2012; Liu et al., 2018; Xiao et al., 2021).

While this study utilizes a global dataset to analyze mSTM ST34, several limitations in our study need to be mentioned. One significant limitation is that our regional surveillance is confined to Zhejiang Province, China. This geographic restriction means that the results may not fully represent the clinical characteristics of mSTM ST34 in other regions or countries, where varying environmental, climatic, and socio-economic factors may influence this pandemic pathogen (Wang et al., 2024). Secondly, our analyses and experiments were limited to three isolates, which may affect the generalizability of our findings. Some of the observed phenotypes might arise from strains isolated from specific hosts. Future studies incorporating data from multiple regions and various sources are essential for providing a more comprehensive understanding of the pathogenicity of mSTM ST34.

In summary, the rapid adaptability, enhanced virulence, and increasing antibiotic resistance of mSTM ST34 pose a continuous public health threat that demands greater attention. More studies and collaborative projects are needed to conduct comprehensive genetic characterization and determine the molecular basis for the success of this pathogen. Enhancing surveillance and reporting systems is crucial to ensure timely detection and documentation of mSTM ST34 cases across healthcare settings. This should be accompanied by improved antibiotic stewardship policies to promote the responsible use of antibiotics, thereby reducing the emergence of multidrug-resistant strains. Furthermore, public health education initiatives are essential to raise awareness about the symptoms and prevention of mSTM ST34, particularly in high-risk populations. Finally, developing emergency response plans and supporting ongoing research into the epidemiological characteristics of mSTM ST34 will be critical for controlling its spread and mitigating its impact on public health.

## Data Availability

The datasets presented in this study can be found in online repositories. The names of the repository/repositories and accession number(s) can be found in the article/**Supplementary Material**.
