# Supplementary material for: Genomic epidemiology and public health implications of zoonotic monophasic Salmonella Typhimurium ST34
Source: Front Cell Infect Microbiol. 2025 Mar 11;15:1490183. doi: 10.3389/fcimb.2025.1490183 (PMC11933091; doi:10.3389/fcimb.2025.1490183)
Supplement: Supplementary file 1 [file Table1.docx]

Supplementary Material

# Supplementary Data

Supplementary Table 1: Brief information about the *Salmonella* isolates globally;

Supplementary Table 2: Brief information about the *Salmonella* strains isolated from patients in this study.
